# Mitochondrial complex I: the key to sustained microglia activation and neuroinflammation maintenance

**DOI:** 10.1186/s40779-024-00554-3

**Published:** 2024-07-19

**Authors:** Hua Wang, Sheng-Yuan Yu, Sofus Nielsen, Xing Wang, Wei-Wei Zhao

**Affiliations:** 1 Key Laboratory of Colloid and Interface Chemistry of the Ministry of Education, School of Chemistry and Chemical Engineering, Shandong University, Jinan, 250100 China; 2 Department of Neurology, Chinese PLA General Hospital, Beijing, 100853 China; 3 Department of Biology, University of Copenhagen, 2100 Copenhagen O, Denmark; 4 Beijing National Laboratory for Molecular Sciences, Institute of Chemistry, Chinese Academy of Sciences, Beijing, 100190 China; 5 Department of Biomaterials, School and Hospital of Stomatology, Cheeloo College of Medicine, Shandong University & Shandong Key Laboratory of Oral Tissue Regeneration & Shandong Engineering Research Center of Dental Materials and Oral Tissue Regeneration & Shandong Provincial Clinical Research Center for Oral Diseases, Jinan, 250012 China

**Keywords:** Mitochondrial complex I, Neuroinflammation, Multiple sclerosis, Reverse electron transport, Microglial activation

Multiple sclerosis (MS) is characterized by chronic, slowly expanding lesions with the accumulation of myeloid cells, which lead to brain atrophy and progressive disability. The role of mitochondria, especially mitochondrial respiratory complexes and metabolites, in controlling myeloid immune responses, is well-documented but not fully understood in diseases of the central nervous system (CNS). The groundbreaking study by Prof. Peruzzotti-Jametti et al. [1], entitled “Mitochondrial complex I activity in microglia sustains neuroinflammation” published in *Nature*, delves into the intricate dynamics between mitochondrial function within microglia and the perpetuation of chronic neuroinflammation, specifically in MS. The core point of their investigation is the hypothesis that mitochondrial complex I (CI) activity, through a mechanism known as reverse electron transport (RET), generates reactive oxygen species (ROS) in microglia, thereby sustaining inflammatory response in the CNS. This increases ROS production from the mitochondria, which is thought to be a crucial factor in the maintenance of a pro-inflammatory state in the microglia, contributing to the pathology of MS and similar neuroinflammatory diseases.

CI, as the largest enzyme in the mitochondrial respiratory chain, primarily function in the electron transport chain is to facilitate ATP production through oxidative phosphorylation [2]. However, when chronic inflammation changes CI’s activity toward RET, the resulting oxidative stress can promote neuronal damage and inhibit the remyelination process, a critical recovery process in MS [3]. The continuous activation of microglia caused by CI results in the accumulation of myeloid cells at lesion sites, further contributing to CNS pathology and symptom severity. Utilizing a combination of single-cell RNA sequencing and metabolic profiling, the authors identified a distinct transcriptional and metabolic signature associated with pro-inflammatory microglia, characterized by enhanced CI activity. Additionally, the metabolic changes suggested a pivotal role of oxidative stress in microglia for sustaining the state of chronic inflammation.

Persistently activated myeloid cells, including microglia, are identified at the edges of chronic lesions in MS. These cells contribute to the formation and maintenance of chronic active lesions that are characteristic of the progressive stages of MS. These cells are also a source of neurotoxic factors, such as tumor necrosis factor, interleukin-1β, nitric oxide, and ROS, which are implicated in remyelination failure and subsequent neuronal and axonal damage [4]. This ongoing impairment is a significant driver of clinical progression and increased disability in people with MS. The authors used single-cell RNA sequencing to reveal the unique transcriptional profiles of microglia from experimental autoimmune encephalomyelitis mice at different stages of the disease, illustrating their transition to a pro-inflammatory phenotype. In addition, imaging and pathological analysis correlated the presence and density of these activated cells with significant areas of neuronal damage and clinical symptoms.

The in vitro experiments confirmed that blocking RET in pro-inflammatory rodent and human microglia, by inhibiting CI activity, effectively prevents excessive extraosseous ROS production and inhibits neurotoxicity [5]. Inhibiting CI offers a dual benefit in the therapeutic context. First, as CI tends to shift activity toward RET in the transition to chronic inflammation, inhibition at this stage might decelerate mitochondrial overproduction of ROS. Second, by altering mitochondrial function, CI inhibition can shift the metabolic profile of immune cells such as microglia and macrophages from a pro-inflammatory to a more neuroprotective or less activated state. The therapeutic implications of CI inhibition are promising, offering a new avenue for the development of treatments for diseases that currently have limited options. The ability to modulate a fundamental aspect of cell metabolism could lead to treatments that are more effective and have fewer side effects than current immunomodulatory therapies.

The study by Peruzzotti-Jametti et al. [1] leaves some questions unanswered. The regulatory mechanisms controlling CI activity in microglia, for example, are still not fully understood. It is essential to identify the upstream signals and transcription factors that modulate CI expression and activity. For instance, are there specific upstream signals or transcription factors that can influence the expression and activity of CI? How do these regulatory factors function in neuroinflammation? Understanding the regulatory pathways that control CI activity, including the specific triggers for RET and ROS production, could reveal new targets for therapeutic intervention to modulate CI activity selectively in microglia. Moreover, the impact of mitochondrial CI activity and its metabolic products on cytoplasmic signaling pathways in microglia are not yet fully understood. Specifically, it remains unclear how mitochondrial signals interact with cytoplasmic inflammatory signaling molecules (such as cytokines and chemokines) to sustain and exacerbate neuroinflammation. Further research is needed to investigate these cross-compartmental signaling mechanisms to elucidate how mitochondrial function systematically regulates cellular inflammatory responses.

Building on the molecular signatures identified in this study, subsequent research should focus on discovering reliable biomarkers for early detection and monitoring of neuroinflammatory disease. These biomarkers could help stratify patients based on the metabolic state of their microglia, enabling more personalized treatment approaches. There may be more specific strategies for addressing neurodegenerative diseases related to microglia: 1) the development of selective inhibitors of CI that specifically target microglia, ideally crossing the blood–brain barrier to inhibit CI activity in microglia without affecting other cell types; 2) the creation of agents that enhance the phagocytic activity of microglia, promoting the clearance of amyloid-beta, Tau, or other pathological aggregates; and 3) the improvement of clinical outcomes by reducing the severity of symptoms and delaying disease progression, thereby enhancing the quality of life for patients, reducing the physical disabilities associated with MS, and extending periods of remission.

In summary, the study by Peruzzotti-Jametti et al. [1] marks a significant advancement in our understanding of neuroinflammation and opens new possibilities for therapeutic intervention. By targeting mitochondrial metabolism in microglia, future research holds the promise of developing innovative treatments that could significantly improve the lives of patients with neurodegenerative diseases. The journey from these foundational discoveries to effective clinical therapies will require concerted efforts, but the potential rewards are immense, offering hope for better management and eventual cures for these debilitating conditions.

## Data Availability

Not applicable.

## Abbreviations

MS: Multiple sclerosis
CNS: Central nervous system
CI: Mitochondrial complex I
RET: Reverse electron transport
ROS: Reactive oxygen species
